# Allelic Variants Within the ABO Blood Group Phenotype Confer Protection Against Critical COVID-19 Hospital Presentation

**DOI:** 10.3389/fmed.2021.759648

**Published:** 2022-01-13

**Authors:** Herbert F. Jelinek, Mira Mousa, Nawal Alkaabi, Eman Alefishat, Gihan Daw Elbait, Hussein Kannout, Hiba AlHumaidan, Francis Amirtharaj Selvaraj, Hala Imambaccus, Stefan Weber, Maimunah Uddin, Fatema Abdulkarim, Bassam Mahboub, Guan Tay, Habiba Alsafar

**Affiliations:** ^1^Center for Biotechnology, Khalifa University of Science and Technology, Abu Dhabi, United Arab Emirates; ^2^Department of Biomedical Engineering, College of Engineering, Khalifa University of Science and Technology, Abu Dhabi, United Arab Emirates; ^3^Center of Heath Engineering Innovation, Khalifa University of Science and Technology, Abu Dhabi, United Arab Emirates; ^4^Nuffield Department of Women's and Reproduction Health, Oxford University, Oxford, United Kingdom; ^5^Department of Pediatric Infectious Disease, Sheikh Khalifa Medical City, Abu Dhabi, United Arab Emirates; ^6^Department of Pharmacology, College of Medicine and Health Sciences, Khalifa University of Science and Technology, Abu Dhabi, United Arab Emirates; ^7^Department of Laboratory Medicine Services, Sheikh Khalifa Medical City, Abu Dhabi, United Arab Emirates; ^8^Dubai Health Authority, Rashid Hospital, Dubai, United Arab Emirates; ^9^Division of Psychiatry, Faculty of Health and Medical Sciences, University of Western Australia, Crawley, WA, Australia; ^10^School of Medical and Health Sciences, Edith Cowan University, Joondalup, WA, Australia; ^11^Department of Genetics and Molecular Biology, College of Medicine and Health Sciences, Khalifa University of Science and Technology, Abu Dhabi, United Arab Emirates

**Keywords:** ABO blood group, SARS-CoV-2, disease severity, UAE, COVID-19 pandemic, infection–immunology, Middle East

## Abstract

**Introduction:** Coronavirus disease 2019 (COVID-19) disease severity differs widely due to numerous factors including *ABO* gene-derived susceptibility or resistance. The objective of this study was to investigate the association of the ABO blood group and genetic variations of the *ABO* gene with COVID-19 severity in a heterogeneous hospital population sample from the United Arab Emirates, with the use of an epidemiological and candidate gene approach from a genome-wide association study (GWAS).

**Methods:** In this cross-sectional study, a total of 646 participants who tested positive for severe acute respiratory syndrome coronavirus 2 (SARS-CoV-2) were recruited from multiple hospitals and population-based (quarantine camps) recruitment sites from March 2020 to February 2021. The participants were divided into two groups based on the severity of COVID-19: noncritical (*n* = 453) and critical [intensive care unit (ICU) patients] (*n* = 193), as per the COVID-19 Reporting and Data System (CO-RADS) classification. The multivariate logistic regression analysis demonstrated the association of ABO blood type as well as circulating anti-A antibodies and anti-B antibodies as well as A and B antigens, in association with critical COVID-19 hospital presentation. A candidate gene analysis approach was conducted from a GWAS where we examined 240 single nucleotide polymorphisms (SNPs) (position in chr9*:* 136125788-136150617) in the *ABO* gene, in association with critical COVID-19 hospital presentation.

**Results:** Patients with blood group O [odds ratio (OR): 0.51 (0.33, 0.79); *p* = 0.003] were less likely to develop critical COVID-19 symptoms. Eight alleles have been identified to be associated with a protective effect of blood group O in *ABO* 3'untranslated region (UTR): rs199969472 (*p* = 0.0052), rs34266669 (*p* = 0.0052), rs76700116 (*p* = 0.0052), rs7849280 (*p* = 0.0052), rs34039247 (*p* = 0.0104), rs10901251 (*p* = 0.0165), rs9411475 (*p* = 0.0377), and rs13291798 (*p* = 0.0377).

**Conclusion:** Our findings suggest that there are novel allelic variants that link genetic variants of the *ABO* gene and ABO blood groups contributing to the reduced risk of critical COVID-19 disease. This study is the first study to combine genetic and serological evidence of the involvement of the ABO blood groups and the *ABO gene* allelic associations with COVID-19 severity within the Middle Eastern population.

## Introduction

Coronavirus disease (COVID-19), caused by the severe acute respiratory syndrome coronavirus 2 (SARS-CoV-2), has spread rapidly worldwide (1). Disease severity differs widely due to numerous factors including SARS-CoV-2 variants and viral load, presence of chronic disease comorbidity, genetic factors, and age among others as well as oxidative stress and inflammation (2–6). The *ABO* gene-derived susceptibility or resistance and ABO blood group antigens have also been associated to COVID-19 disease severity (7). Age has been well described as a risk factor with the 0–4 years age group being 4 times less likely and the 85+ age group being 13 times more likely to be hospitalized compared to the 18–29 age groups (3). Comorbidities including obesity, diabetes, cardiovascular disease, chronic kidney disease, hypertension, tuberculosis, respiratory disease, inflammatory disease, coagulation dysfunction, and cancer have been reported to play a role in disease severity (4). Timing to hospital admission, challenges on health systems and expertise in healthcare delivery are also associated with the risk of increased progression in noncommunicable and communicable diseases including progression to severe COVID-19 presentation requiring hospitalization (8).

The ABO blood typing and the *ABO* gene variant analysis have been a central component of the immune response in transplantation and transfusion medicine, but has also been of importance in the immune response and progression of viral infections (9). The ABO blood system consists of different molecular determinants leading to different antigenic structures that play varying roles in the immune response. The A and B alleles encode different glycosyltransferases that add N-acetylgalactosamine and D-galactose to the common precursor H determinant side chain, which converts to A or B antigen. As O alleles do not encode a functional enzyme due to a premature stop codon within the glycosyltransferase gene yielding a truncated gene, it results in a complete loss of enzymatic activity and unchanged H-antigen (10, 11).

The genetic basis of the human ABO blood groups is located on chromosome 9 (9q34.1) and is associated with the synthesis of specific ABO glycosyltransferases (12). The ABO blood groups have been included in disease risk and severity of COVID-19, although with mixed results (13). The first link between coronavirus infection and ABO was reported in 2005 following the SARS-CoV-2 pandemic (14). These authors reported that health professionals with type O blood group were less likely to be infected compared to those with other blood types. More recent studies on SARS-CoV-2 reported similar associations between viral infection and the ABO blood groups (15, 16). However, divergent results are still coming forth with respect to the role of the ABO blood groups as a risk marker for COVID-19 severity linked to possible comorbidities and methodological differences as well as population differences in tissue receptors associated with SARS-CoV-2 infection (4, 13, 17–19).

### ABO Blood Groups as a Risk Factor in Nonviral Diseases

Determining any link between the ABO blood groups and disease is complicated due to the highly polymorphic nature of the ABO blood group system and, hence, more often related to ABO phenotype rather than ABO genotype (20, 21). The risk of disease associated with a specific blood group has been shown for coronary artery disease, ischemic stroke, cancer, dementia, hypertension, hyperlipidemia, and diabetes (22–25). The ABO blood groups modulate both the hemostasis and endothelial function, leading to interactions with inflammatory cells and a positive association to metabolic and cardiovascular conditions (26, 27). Therefore, when investigating the role of ABO serology and association with COVID-19 severity, the role of ABO blood groups in nonviral pathology is an important factor because severe COVID-19 is characterized as an inflammatory state that damages the alveolar capillary barrier and compromises gas exchange, leading to intracapillary thrombosis and endothelial dysfunction. This is further highlighted by recent findings of increased morbidity and mortality of patients with COVID-19 with comorbidities (28).

### ABO Blood Groups as a Risk Factor in Bacterial and Viral Diseases

Similar to the link between the risk of nonviral diseases, the ABO blood groups have been associated with diverse bacterial and viral infections. Several enteric organisms, including *Vibrio cholera* are linked to the risk of disease and disease severity, where individuals with blood group O have a more severe disease after infection (29). Other infections include mumps, tuberculosis, plague, malaria, and norovirus among others (20, 21, 30, 31). Type O blood has been linked to cholera bacteria and greater susceptibility to infection by *Helicobacter pylori*, plague, and mumps compared to people with either the A or B variants (22, 32, 33). Blood group A increases the risk of smallpox, while blood group B is associated with an increased risk of tuberculosis, *Streptococcus pneumoniae, Escherichia coli*, and *Salmonella* infections (21). Viral infections and association with the ABO blood groups have been reported for influenza, picornaviruses, hepatitis B virus, norovirus, and HIV infection (34–38). The association between the ABO blood group and infectious diseases may be due to modifications of a key target cell surface glycoprotein or glycolipid, thereby affecting important cellular functions, such as endocytosis, phagocytosis, and signal transduction, in response to infection (16).

### ABO Blood Groups as a Risk Factor in COVID-19

Coto et al. conducted a large meta-analysis investigating the susceptibility or protection of the ABO blood groups associated with SARS-CoV-2 of over 30,000 cases. This study was not able to determine a link between the ABO blood group and severity or mortality associated with COVID-19 (39). Although a 45% higher risk of developing COVID-19, if the A blood type was present and a 35% lower risk with type O blood reported by others (2). More recent studies of available data have drawn similar conclusions highlighting the necessity to account for potential confounders (16).

### ABO Genetic Association Studies and COVID-19

The gene locus for the human ABO blood groups is located on chromosome 9 (9q34.1) and is associated with the synthesis of specific ABO glycosyltransferases (12). The *ABO* glycosyltransferase gene also presents with common polymorphisms including a one nucleotide deletion in exon 6 (codon 87) determining the O allele and four single nucleotide polymorphisms (SNPs) at residues 176, 235, 266, and 268, which switch enzyme function from A transferase to B transferase activity (12, 40). Preliminary results from the 23andMe consortium of genome sequencing have indicated a protective effect of group O and reported that the rs505922 SNP in the *ABO* gene with a T substitution at that location is associated with a lower risk of SARS-CoV-2 infection. Similar findings were reported for individuals with the type O blood group who were less likely to be infected, whereas those individuals with the type A blood group were more likely to be infected (13, 41). Genome data from Europe and Australia confirmed these findings. In COVID-19 genome-wide association study (GWAS), the authors studied a cohort in Italy and Spain, suggesting a correlation between the ABO blood groups and SARS-CoV-2 susceptibility (2). This cohort included 835 patients with severe COVID-19 who were hospitalized with respiratory failure and 1,255 control participants from Italy and 775 patients and 950 control participants from Spain. The final analysis showed significant associations with rs657152 at locus 9q34.2, which is in almost complete linkage disequilibrium (LD) with rs8176719 of the *ABO* blood group locus, the main determinant of the group O, allele ABO^*^O.01.01 (2). In contrast, a Danish study reported no association of rs657152 with COVID-19 infection or outcome (42). Data from other studies participating in the COVID-19 Host Genetics Initiative combined with the data from COVID-19 GWAS also found no association with the ABO blood group locus (43).

Therefore, further investigation and study are warranted to clarify the relationship between COVID-19 and the *ABO* gene variants, especially with respect to regional differences. Khalil et al. (44) analyzing the ABO blood group associations with COVID-19 in a Middle East and North African cohort could not corroborate previous studies that indicated a link between the ABO blood groups and susceptibility to or severity of SARS-CoV-2 infection. Only a limited number of genetic studies have addressed COVID-19 severity and comparing critical to noncritical in hospital patients with conflicting results and not with respect to *ABO* gene polymorphisms (15, 45, 46). One study provided results for single polymorphism and COVID-19 infection (2). Therefore, the aim of this study is to ascertain whether the specific ABO blood group is associated with COVID-19 severity and identify allelic variants on the *ABO* gene that is associated with disease severity in a heterogeneous population sample from the United Arab Emirates (UAE).

## Methods

### Study Participants and Recruitment

Patients with COVID-19 were recruited from multiple recruitment sites (hospitals and facility quarantine sites) across the UAE. Only patients who tested positive for SARS-CoV-2 by real-time PCR (RT-PCR) were included in this study cohort. The participants were divided into two groups based on the severity of COVID-19, which was indicated by the treating physician as noncritical (*n* = 453) or critical (*n* = 193) based on the COVID-19 Reporting and Data System (CO-RADS) classification (47). In brief, participants were defined as critical COVID-19 cases, if they are admitted to the intensive care unit (ICU) with the use of oxygen supplementation or mechanical ventilation. An informed written consent form was obtained in accordance with the Declaration of Helsinki. This study was approved by the Abu Dhabi Health COVID-19 Research Ethics Committee (DOH/DQD/2020/538), the Dubai Scientific Research Ethics Committee (DSREC-04/2020_09), and the SEHA Research Ethics Committee (SEHA-IRB-005). Consent was obtained from a family member of the patients who were on ventilators, with the approval of a supervising physician.

### Sample Collection

A total of 2 ml blood samples of 646 patients with COVID-19 were collected in a sterile 5-ml sample tube supplemented with ethylenediaminetetraacetic acid (EDTA) from the cubital vein by experienced venipuncture nurses. Samples were transported in a sealed biohazard bag using a cool transport container to Khalifa University Center for Biotechnology in Abu Dhabi. COVID-19 infection was confirmed by a RT-PCR test of SARS-CoV-2.

### Deoxyribonucleic Acid Extraction and Genotyping

Deoxyribonucleic acid was extracted using the automated MagPurix 12 system according to the protocol of the manufacturer. DNA was quantified using the DS-11 Series of Spectrophotometer/Fluorometer (DeNovix, Wilmington, Delaware, USA). Genotyping was performed using the Infinium Global Screening Array (Illumina Incorporation, San Diego, California, USA), which contained around 654,027 genetic markers and developed by Avera Institute for Huma Genetics (Sioux Falls, South Dakota, USA). Quality control (QC) on the data was performed using the PLINK software (version 1.07) to exclude SNPs with a low minor allele frequency (<0.01), low genotyping rate (<95%), and deviation from Hardy–Weinberg equilibrium (p < 10^−4^) significance level. Samples that failed to reach 98.5% call rate were reanalyzed. After quality control, 417,263 variants passed filters. Participant genotype data were phased and imputed using the Phase 3 1000 Genomes Projects panel (https://mathgen.stats.ox.ac.uk/impute/1000GP_Phase3.html). Variants with low imputation quality (r^2^ < 0.5 averaged across the batch) were removed. A total of 240 SNPs (position in chr9*:* 136,125,788–136,150,617) in the *ABO* gene located at chromosome 9q34.2 were extracted for the association of this study for candidate gene analyses.

### Statistical Analysis

The statistical analysis was conducted as a case–control panel, with controls characterized as noncritical symptoms and cases characterized as critical symptoms, with the use of the PLINK software (version 1.9), R software (version 3.4), and the SPSS software (version 16.0). The chi-squared test was used to study categorical variables by cross-tabulation. The bivariate and multivariate logistic regression analyses were used to estimate the odds ratio (OR) and *p*-values of the association between blood type and COVID-19 severity phenotypes. All the regression models accounted for gender (male and female), age (continuous), current body mass index (BMI) (continuous), and presence of comorbidities (yes and no). Comorbidities were defined as a previous medical diagnosis of diabetes mellitus, hypertension, cardiac disease, lung disease, liver disease, kidney disease, metabolic disorder, and/or an autoimmune disease. For candidate gene analysis, we examined 240 SNPs (position in chr9*:* 136125788–136150617) in the *ABO* gene. Two candidate gene association tests were conducted that included unadjusted analysis and adjustment on the top ten eigenvectors for population stratification, age, and gender. We tested for association using logistic regression, assuming additive allelic effects. The significance level adopted for all the analyses was *p* ≤ 0.05. For the significant SNPs, we evaluated the frequency of genotype and alleles by stratifying based on the ABO blood group.

## Results

A total of 646 participants were included in the cohort, with 453 participants admitted to hospital for noncritical COVID-19 and 193 participants admitted for critical COVID-19 (Table 1). The average age was 44.70 years (SD ± 15.53) and average BMI was 28.11 kg/m^2^ (SD ± 15.53). Males (78.6%) and Asian nationality (57.0%) made up the majority of the overall population. The noncritical COVID-19 group was significantly younger (*p* < 0.001) and contained the lower BMI group (*p* = 0.001). Gender was not associated with a critical presentation of COVID-19 disease (*p* = 0.640).

**Table 1:** Demographic characteristics.

	**Non-critical**	**Critical**	***p*-value**
	**(*n* = 453)**	**(*n* = 193)**	
**Gender**			
Female	99 (21.9%)	39 (20.2%)	0.640
Male	354 (78.1%)	154 (79.8%)	
**Age**			
1–29	98 (21.6%)	4 (2.1%)	<0.001
30–38	135 (29.8%)	23 (11.9%)	
39–49	112 (24.71%)	41 (21.2%)	
50–85	108 (23.8%)	125 (64.7%)	
BMI^*^			
≤ 18.5	9 (2.3%)	1 (0.5%)	0.001
>18.5 to ≤ 24.9	115 (29.4%)	38 (19.7%)	
>24.9 to ≤ 29.9	168 (43.0%)	77 (39.9%)	
>29.9	99 (25.3%)	77 (39.9%)	
**Region of origin**			
Middle East	161 (35.5%)	75 (38.9%)	0.002
Asia	273 (60.3%)	95 (49.2%)	
Africa	13 (2.9%)	16 (8.3%)	
Europe	4 (0.9%)	3 (1.6%)	
America	2 (0.4%)	4 (2.1 %)	
Past medical history^*^			
Yes	141 (31.1%)	111 (57.5%)	<0.001
No	312 (68.9%)	82 (42.5%)	

* *Past medical history includes a past medical diagnosis of one or more of the following comorbid conditions: diabetes, cardiovascular disease, chronic kidney disease, hypertension, tuberculosis, respiratory disease, inflammatory disease, coagulation dysfunction, and cancer*.

Table 2 demonstrates the prevalence and logistic regression analysis of the ABO blood type characteristics of a subset of the participants (*n* = 527). The multivariate regression analysis adjusted for age, gender, BMI, and presence of comorbid conditions demonstrated that patients with a blood group B [OR: 0.97 (0.57, 1.65); *p* = 0.975] and blood group AB [OR: 0.61 (0.25, 1.46): *p* = 0.273] had no association with critical COVID-19. However, there was a protective effect for blood group O [OR: 0.51 (0.31, 0.84); *p* = 0.008] from developing critical COVID-19, which is consistent with previous findings. After adjustment, circulating anti-A antibodies and anti-B antibodies, as well as A and B antigens, in the plasma had no association to COVID-19 critical phenotype. Given that age and BMI play a significant role on COVID-19 severity, stratification of the blood group was conducted (Supplementary Table 1), demonstrating that age plays a significant role in COVID-19 severity.

**Table 2:** Association of blood type characteristics to noncritical vs. critical coronavirus disease 2019 (COVID-19) hospital presentation.

	**Prevalence (%)**			**Unadjusted**		Adjusted^*^	
	**Non-critical (n = 341)**	**Critical (n = 186)**	***p*-value**	**Odds ratio (95% CI)**	***p*-value**	**Odds ratio (95% CI)**	***p*-value**
**Blood type**			0.007				
A	95 (27.9%)	65 (34.9%)		1.39 (0.95, 2.04)	0.091	1.46 (0.95, 2.25)	0.086
B	77 (22.6%)	57 (30.6%)		1.52 (1.00, 2.26)	0.048	1.50 (0.96, 1.65)	0.068
AB	23 (6.7%)	12 (6.5%)		0.95 (0.46, 1.96)	0.897	0.78 (0.35, 1.81)	0.575
O	146 (42.8%)	52 (28.0%)		0.52 (0.33, 0.76)	0.001^**^	0.51 (0.33, 0.79)	0.003^**^
**Anti-A antibodies**			0.090		0.091		0.080
No	95 (29.9%)	65 (37.4%)		1.00		1.00	
Yes	223 (70.1%)	109 (62.6%)		0.71 (0.48, 1.05)		0.67 (0.43, 1.05)	
**Anti-B antibodies**			0.042		0.042		0.163
No	77 (24.2%)	57 (32.8%)		1.00		1.00	
Yes	241 (75.8%)	117 (67.2%)		0.65 (0.43, 0.98)		0.72 (0.45, 1.14)	
**A antigen**			0.123		0.123		0.144
No	223 (65.4%)	109 (58.6%)		1.00		1.00	
Yes	118 (34.6%)	77 (41.4%)		1.33 (0.92, 1.92)		1.36 (0.89, 2.06)	
**B antigen**			0.068		0.068		0.275
No	241 (70.7%)	117 (62.9%)		1.00		1.00	
Yes	100 (29.3%)	69 (37.1%)		1.42 (0.97, 2.07)		1.27 (0.82, 1.95)	
**Rh factor**			0.706		0.706		0.817
Positive	316 (92.7%)	174 (93.5%)		1.00		1.00	
Negative	25 (7.3%)	12 (6.5%)		1.14 (0.56, 2.34)		1.09 (0.49, 2.43)	

* *Adjusted for age, gender, body mass index (BMI), and presence of comorbid conditions;*

** *significant association*.

To investigate if COVID-19 severity was associated with specific SNPs located on the *ABO* locus, all the 240 SNPs located in the *ABO* candidate gene were extracted from a GWAS and analyzed in this study. The *ABO* candidate gene consists of a 5′-untranslated region (UTR), seven exons, and 3′-UTR. Supplementary Table 1 demonstrates the unadjusted and adjusted OR and *p*-value of the SNPs at the *ABO* locus. As blood type frequencies vary across the ancestry groups, the confounding effect of ancestry was evaluated by adjusting for ethnicity. The first ten eigenvectors were used in subsequent analyses for adjustment to population stratification. Following these adjustments, eight SNPs were identified on the *ABO* gene locus that was associated with COVID-19 severity, all located in the 3'-UTR region (Table 3).

**Table 3:** Unadjusted and adjusted effect size and *p*-value of the eight significant single nucleotide polymorphisms (SNPs) in chromosome 9 on the *ABO* gene.

		**Unadjusted**		Adjusted^*^	
**SNP**	**Effect allele**	**Odds ratio**	***p*-value**	**Odds ratio**	***p*-value**
rs199969472	A	1.53 (1.11, 2.11)	0.0089	1.77 (1.18, 2.66)	0.0052
rs34266669	T	1.53 (1.11, 2.11)	0.0089	1.77 (1.18, 2.66)	0.0052
rs76700116	C	1.53 (1.11, 2.11)	0.0089	1.77 (1.18, 2.66)	0.0052
rs7849280	G	1.52 (1.10, 2.08)	0.0105	1.77 (1.18, 2.66)	0.0052
rs34039247	C	1.72 (1.09, 2.73)	0.0178	2.35 (1.22, 4.55)	0.0104
rs10901251	C	1.44 (1.13, 1.85)	0.0032	1.45 (1.07, 1.96)	0.0165
rs9411475	C	1.39 (1.00, 1.94)	0.0493	1.55 (1.03, 2.36)	0.0377
rs13291798	G	1.47 (0.87, 2.48)	0.1439	2.08 (1.03, 4.22)	0.0415

* *Adjusted for age, gender, and population stratification*.

**Figure 1:**
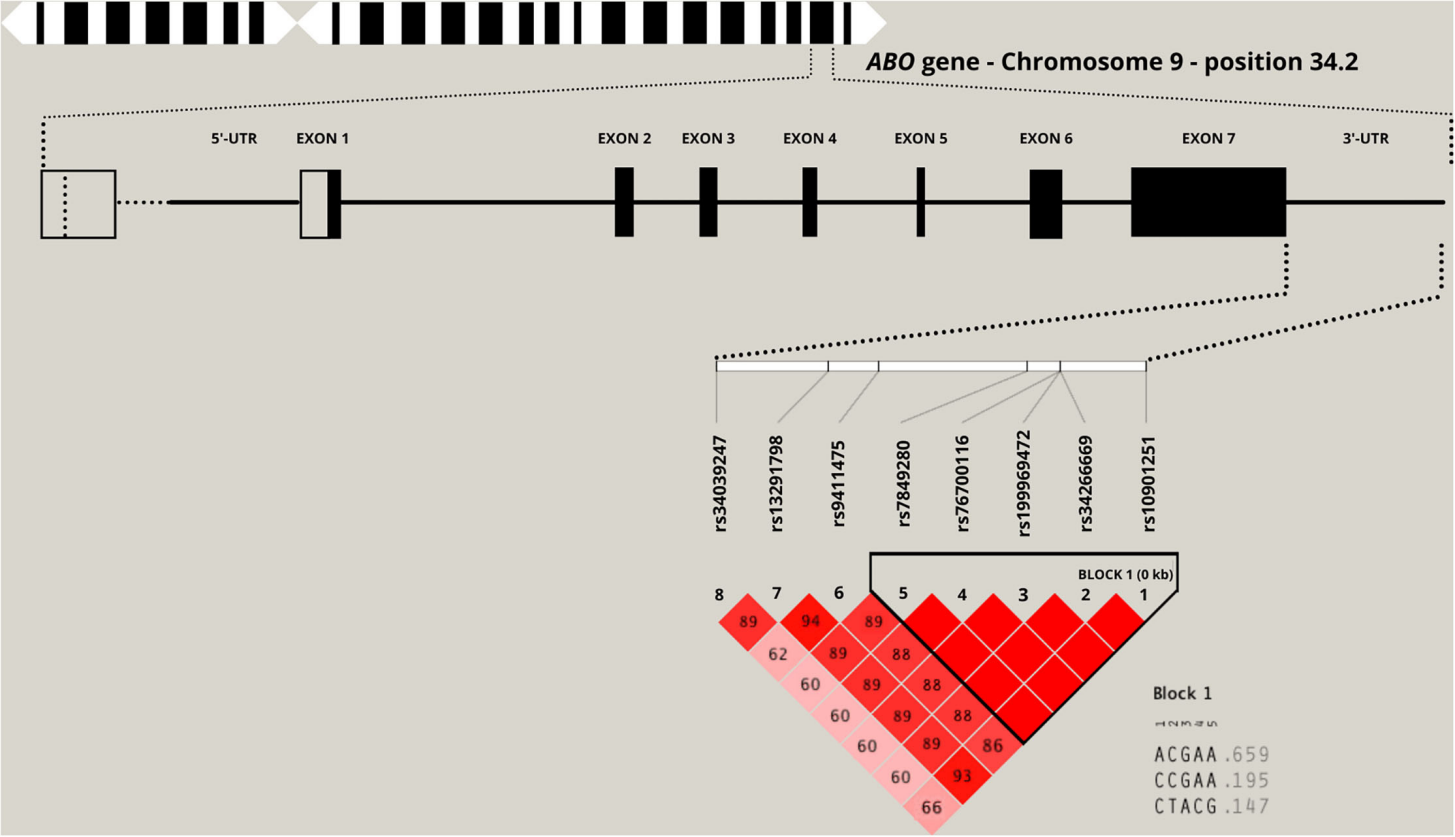
Location of the top eight significant single nucleotide polymorphisms (SNPs) in the 3-untranslated region (3'-UTR) of the *ABO* candidate gene. The numbers inside each square and the square shading indicate the degree of linkage disequilibrium (LD) between SNPs (complete LD, D′ = 1.0 and no LD, D′ = 0.0).

An upregulation of the following SNPs, located in the same haplotype block (D′ = 1.0; as given in Figure 1), is associated with the critical COVID-19 phenotype: rs199969472 [OR: 1.77 (95% CI: 1.18, 2.66); *p* = 0.0052], rs10901251 [OR: 1.45 (95% CI: 1.07, 1.96), *p* = 0.0165], rs34266669 [OR: 1.77 (95% CI: 1.18, 2.66); *p* = 0.0052], rs76700116 [OR: 1.77 (95% CI: 1.18, 2.66); *p* = 0.0052], and rs7849280 [OR: 1.77 (95% CI: 1.18, 2.66); *p* = 0.0052]. Haplotype analyses (Figure 1 and Table 4) of the 5 SNPs were performed to estimate the genetic contribution of haplotypes to critical COVID-19 phenotype, demonstrating a significant association to two haplotype blocks (ACGAA, *p* = 0.0028; CTACG, *p* = 0.0079). SNP rs34039247 [OR: 2.35 (95% CI: 1.22, 4.55); *p* = 0.0104], SNP rs9411475 [OR: 1.55 (95% CI: 1.03, 2.36); *p* = 0.0377], and SNP rs13291798 [OR: 1.55 (1.03, 2.36); *p* = 0.0377] have also been identified to be associated with critical COVID-19 phenotype.

**Table 4:** Association analyses of the haplotype block with critical COVID-19 hospital presentation.

**Block 1**	**Haplotype**	**Case, control frequencies**	***p-*value**
ACGAA	0.659	0.598, 0.685	0.0028
CCGAA	0.195	0.215, 0.186	0.2253
CTACG	0.147	0.187, 0.129	0.0079

To determine which of these alleles contribute to the protective effect found for blood group O in the current cohort, the genotype and allele frequencies of the eight significant SNPs were stratified according to blood group (Table 5). The effect alleles of the eight SNPs associated with higher risk of COVID-19 severity (Table 3) had a significantly lower frequency in patients with blood group O in noncritical vs. critical patients. Of interest is that this is the first report of an allele being identified that is associated with a protective effect of blood group O. For the SNP rs19996947, the A allele was found to be present in 28.8% of critical cases vs. the G allele, which was present in 71.2% of the cases, indicating a significant association (*p* = 0.002).

**Table 5:** Genotype and allelic stratification of eight significant SNPs in chromosome 9 on the *ABO* gene into the ABO blood group.

**SNP**	**Blood group**	**Genotype**	**Non-critical (*n* = 341)**	**Critical (*n* = 186)**	***p*-value**	**Allele**	**Non-critical (*n* = 341)**	**Critical (*n* = 186)**	***p*-value**
rs199969472	A	GG	46 (48.4%)	26 (40.0%)	0.575	G	46 (48.4%)	26 (40.0%)	0.293
		GA	44 (46.3%)	35 (53.8%)		A	49 (51.6%)	39 (60.0%)	
		AA	5 (5.3%)	4 (6.2%)					
	AB	GG	11 (47.8%)	6 (50.0%)	0.349	G	11 (47.8%)	6 (50.0%)	0.903
		GA	12 (52.2%)	5 (41.7%)		A	12 (52.2%)	6 (50.0%)	
		AA	0 (0.0%)	1 (8.3%)					
	B	GG	69 (89.6%)	53 (93.0%)	0.295	G	69 (89.6%)	53 (93.0%)	0.499
		GA	8 (10.4%)	3 (5.3%)		A	8 (10.4%)	4 (7.0%)	
		AA	0 (0.0%)	1 (1.8%)					
	O	GG	130 (89.0%)	37 (71.2%)	0.005	G	130 (89.0%)	37 (71.2%)	0.002
		GA	15 (10.3%)	15 (28.8%)		A	16 (11.0%)	15 (28.8%)	
		AA	1 (0.7%)	0 (0.0%)					
rs34266669	A	CC	46 (48.4%)	26 (40.0%)	0.575	C	46 (48.4%)	26 (40.0%)	0.293
		CT	44 (46.3%)	35 (53.8%)		T	49 (51.6%)	39 (60.0%)	
		TT	5 (5.3%)	4 (6.2%)					
	AB	CC	11 (47.8%)	6 (50.0%)	0.349	C	11 (47.8%)	6 (50.0%)	0.903
		CT	12 (52.2%)	5 (41.7%)		T	12 (52.2%)	6 (50.0%)	
		TT	0 (0.0%)	1 (8.3%)					
	B	CC	69 (89.6%)	53 (93.0%)	0.295	C	69 (89.6%)	53 (93.0%)	0.499
		CT	8 (10.4%)	3 (5.3%)		T	8 (10.4%)	4 (7.0%)	
		TT	0 (0.0%)	1 (1.8%)					
	O	CC	130 (89.0%)	37 (71.2%)	0.005	C	130 (89.0%)	37 (71.2%)	0.002
		CT	15 (10.3%)	15 (28.8%)		T	16 (11.0%)	15 (28.8%)	
		TT	1 (0.7%)	0 (0.0%)					
rs76700116	A	AA	46 (48.4%)	26 (40.0%)	0.575	A	46 (48.4%)	26 (40.0%)	0.293
		AC	44 (46.3%)	35 (53.8%)		C	49 (51.6%)	39 (60.0%)	
		CC	5 (5.3%)	4 (6.2%)					
	AB	AA	11 (47.8%)	6 (50.0%)	0.349	A	11 (47.8%)	6 (50.0%)	0.903
		AC	12 (52.2%)	5 (41.7%)		C	12 (52.2%)	6 (50.0%)	
		CC	0 (0.0%)	1 (8.3%)					
	B	AA	69 (89.6%)	53 (93.0%)	0.295	A	69 (89.6%)	53 (93.0%)	0.499
		AC	8 (10.4%)	3 (5.3%)		C	8 (10.4%)	4 (7.0%)	
		CC	0 (0.0%)	1 (1.8%)					
	O	AA	130 (89.0%)	37 (71.2%)	0.005	A	130 (89.0%)	37 (71.2%)	0.002
		AC	15 (10.3%)	15 (28.8%)		C	16 (11.0%)	15 (28.8%)	
		CC	1 (0.7%)	0 (0.0%)					
rs7849280	A	AA	46 (48.4%)	26 (40.0%)	0.551	A	46 (48.4%)	26 (40.0%)	0.293
		AG	43 (45.3%)	35 (53.8%)		G	49 (51.6%)	39 (60.0%)	
		GG	6 (6.3%)	4 (6.2%)					
	AB	AA	11 (47.8%)	6 (50.0%)	0.349	A	11 (47.8%)	6 (50.0%)	0.903
		AG	12 (52.2%)	5 (41.7%)		G	12 (52.2%)	6 (50.0%)	
		GG	0 (0.0%)	1 (8.3%)					
	B	AA	69 (89.6%)	53 (93.0%)	0.295	A	69 (89.6%)	53 (93.0%)	0.499
		AG	8 (10.4%)	3 (5.3%)		G	8 (10.4%)	4 (7.0%)	
		GG	0 (0.0%)	1 (1.8%)					
	O	AA	130 (89.0%)	37 (71.2%)	0.005	A	130 (89.0%)	37 (71.2%)	0.002
		AG	15 (10.3%)	15 (28.8%)		G	16 (11.0%)	15 (28.8%)	
		GG	1 (0.7%)	0 (0.0%)					
rs34039247	A	AA	80 (84.2%)	51 (78.5%)	0.650	A	80 (84.2%)	51 (78.5%)	0.354
		AC	14 (14.7%)	13 (20.0%)		C	15 (15.8%)	14 (21.5%)	
		CC	1 (1.1%)	0 (0.0%)					
	AB	AA	19 (82.6%)	11 (91.7%)	0.467	A	19 (82.6%)	11 (91.7%)	0.467
		AC	4 (17.4%)	1 (8.3%)		C	4 (17.4%)	1 (8.3%)	
		CC	0 (0.0%)	0 (0.0%)					
	B	AA	74 (98.7%)	55 (96.5%)	0.493	A	76 (98.7%)	55 (96.5%)	0.393
		AC	3 (1.3%)	1 (1.8%)		C	1 (1.3%)	2 (3.5%)	
		CC	0 (0.0%)	1 (1.8%)					
	O	AA	139 (93.2%)	41 (78.8%)	0.012	A	136 (93.2%)	41 (78.8%)	0.004
		AC	7 (4.8%)	9 (17.3%)		C	10 (6.8%)	11 (21.2%)	
		CC	3 (2.1%)	2 (3.8%)					
rs10901251	A	AA	39 (41.1%)	25 (38.5%)	0.597	A	39 (41.1%)	25 (38.5%)	0.742
		AC	50 (52.6%)	33 (50.8%)		C	56 (58.9%)	40 (51.5%)	
		CC	6 (6.3%)	7 (10.8%)					
	AB	AA	1 (4.3%)	0 (0.0%)	0.740	A	1 (4.3%)	0 (0.0%)	0.464
		AC	10 (43.5%)	6 (50.0%)		C	22 (95.7%)	12 (100.0%)	
		CC	12 (52.2%)	6 (50.0%)					
	B	AA	8 (10.4%)	2 (3.5%)	0.320	A	8 (10.4%)	2 (3.5%)	0.134
		AC	53 (68.8%)	43 (75.4%)		C	69 (89.6%)	55 (96.5%)	
		CC	16 (20.8%)	12 (21.2%)					
	O	AA	121 (82.9%)	36 (69.2%)	0.107	A	121 (82.9%)	36 (69.2%)	0.037
		AC	21 (14.4%)	14 (26.9%)		C	25 (17.1%)	16 (30.8%)	
		CC	4 (2.7%)	2 (2.8%)					
rs9411475	A	TT	47 (49.5%)	28 (43.1%)	0.586	T	47 (49.5%)	28 (43.1%)	0.426
		TC	42 (44.2%)	34 (52.3%)		C	48 (50.5%)	37 (56.9%)	
		CC	6 (6.3%)	3 (4.6%)					
	AB	TT	10 (43.5%)	6 (50.0%)	0.713	T	10 (43.5%)	6 (50.0%)	0.713
		TC	13 (56.5%)	6 (50.0%)		C	13 (56.5%)	6 (50.0%)	
		CC	0 (0.0%)	0 (0.0%)					
	B	TT	73 (94.8%)	55 (96.5%)	0.641	T	73 (94.8%)	55 (96.5%)	0.641
		TC	4 (5.2%)	2 (3.5%)		C	4 (5.2%)	2 (3.5%)	
		CC	0 (0.0%)	0 (0.0%)					
	O	TT	129 (88.4%)	38 (73.1%)	0.020	T	129 (88.4%)	38 (73.1%)	0.009
		TC	16 (11.0%)	14 (26.9%)		C	17 (11.6%)	14 (26.9%)	
		CC	1 (0.7%)	0 (0.0%)					
rs13291798	A	AA	83 (87.4%)	53 (81.5%)	0.348	A	83 (87.4%)	53 (81.5%)	0.310
		AG	11 (11.6%)	12 (18.5%)		G	12 (12.6%)	12 (18.5%)	
		GG	1 (1.1%)	0 (0.0%)					
	AB	AA	20 (87.0%)	10 (83.3%)	0.771	A	20 (87.0%)	10 (83.3%)	0.771
		AG	3 (13.0%)	2 (16.7%)		G	3 (13.0%)	2 (16.7%)	
		GG	0 (0.0%)	0 (0.0%)					
	B	AA	74 (96.1%)	56 (98.2%)	0.471	A	74 (96.1%)	56 (98.2%)	0.471
		AG	3 (3.9%)	1 (1.8%)		G	3 (3.9%)	1 (1.8%)	
		GG	0 (0.0%)	0 (0.0%)					
	O	AA	139 (95.2%)	44 (84.6%)	0.021	A	139 (95.2%)	44 (84.6%)	0.013
		AG	6 (4.1%)	8 (15.4%)		G	7 (4.8%)	8 (15.4%)	
		GG	1 (0.7%)	0 (0.0%)					

Five SNPs are in moderate-strong LD with the SNP associated with the blood type O allele (rs8176719; rs199969472, D′ = 0.62; rs10901251, D′ = 0.53; rs76700116, D′ = 0.62; rs7849280, D′ = 0.62; rs9411475, D′ = 0.55). Only two SNPs are in moderate-strong LD with the SNP associated with the blood type A allele (rs8176746; rs10901251, D′ = 0.66; rs34039247, D′ = 0.96) and blood type B allele (rs8176747; rs10901251, D′ = 0.66; rs34039247, D′ = 0.96). Although our data did not demonstrate significant association to the blood type O allele (rs8176719), the SNPs in moderate and strong LD was associated with a significantly reduced risk of critical phenotype presentation with COVID-19.

## Discussion

This study is the first study to combine genetic and serological evidence of the involvement of the ABO blood groups and *ABO* gene allelic associations with COVID-19 severity within a Middle Eastern population investigated in the UAE, a country that is characterized by the convergence of multiple cultural and socioeconomic factors.

Patients with blood group O [OR: 0.51 (0.31, 0.84); *p* = 0.008] were less likely to present with critical COVID-19 to the hospitals included in this study, which is consistent with previous findings. Studies have demonstrated that the adhesion of S protein and angiotensin-converting enzyme 2 (ACE2), a receptor for SARS-CoV-2, can be inhibited by anti-A natural antibody (11). Therefore, the anti-A and anti-B natural antibodies produced by patients with blood group O may decrease the risk of developing critical symptoms by blocking the interaction with ACE2 receptor and prevent viral entry into the lung epithelium. Our data strengthen the evidence for a role for ABO in COVID-19 susceptibility and severity and is notable given the reported links between COVID-19 and blood clotting complications that has now been associated with C5a activity (48).

With respect to the *ABO* locus, eight SNPs showed an association signal in a candidate gene level and play a role in dysregulating the antibody, natural killer cell, and immune mediator profiles: rs199969472 (*p* = 0.0052), rs34266669 (*p* = 0.0052), rs76700116 (*p* = 0.0052), rs7849280 (*p* = 0.0052), rs34039247 (*p* = 0.0104), rs10901251 (*p* = 0.0165), rs9411475 (*p* = 0.0377), and rs13291798 (*p* = 0.0377). Stratification of the SNPs with respect to the ABO blood group indicated significant associations between blood group O and the allelic polymorphism that provided protection to the current cohort for critical presentation of COVID-19. All the SNPs that have showed an association signal are located at 3'-UTR, which play an important role in the *ABO* gene expression and transcriptional signaling in association with COVID-19 symptom presentation (49).

The eight SNPs identified in this study, however, did not include rs657152 and rs8176719, which have been previously observed to be associated with COVID-19 (2). Several reasons can be proposed for this finding including a yet unidentified interaction between different genome variants with minor allele frequencies below 5% and very weak effects (50). Geographical distribution of ancestral risk alleles and population characteristics such as consanguinity, environmental pressures, and incorrect reporting of phenotype may also have contributed to the differences to previous observed results (51). However, the differences in association between the ABO blood group and SNPs may also be explained by the study of Valenti et al. (48) who reported that the association between impact of rs11385949 and the ABO blood group on complement activation disappeared at 30 days after admission during remission of symptoms in surviving patients.

Conflicting findings are present in studies that have investigated the association of the ABO blood group and COVID-19 susceptibility and severity (16, 41, 52–58). Most systematic reviews and meta-analysis studies have demonstrated a decreased risk in susceptibility and severity among patients with COVID-19 in O group (10, 13, 28, 54, 59), whereas other reviews and manuscripts demonstrated no significant association (39, 53, 60–62). These differences may be due to ascertainment bias, multiple confounding effects, diverse studies populations and their geographics locations, presence and adjustment to comorbidities, and case and control selection criteria. These factors may lead to inaccurate estimation of the relative risk. Therefore, appropriately designed observational case–control studies would be more suited to minimize bias and investigate the association of the ABO blood group and COVID-19 severity. In addition, given that the population ancestries in Asian and Middle Eastern countries are relatively homogeneous, relative frequencies of ABO phenotypes may affect the findings of this study. In this study, even with the adjustment of stratification for ancestry, 8 SNPs were identified on the *ABO* gene locus to be significantly associated with COVID-19 severity, with a lower frequency (protective factor) of the effect allele in patients with blood group O.

Direct susceptibility or resistance of disease may be a function of disease severity, as shown by a meta-analysis reported by Pourali et al. (28). In addition, host protein levels may play a role in disease severity in terms of virulence, as blood group O is associated with lower *ACE* levels and the converse has been found for blood group A (27). Associated with this finding is the binding probability of SARS-CoV-2. *ACE2*, being the main receptor, binds to cells expressing blood group moieties most reported in the mucous membrane of the respiratory tract. Thus, blood group AB has the most contact and blood group O the least contact with SARS-CoV-2 (27, 63). This is further demonstrated in this study, where patients with blood type O were less likely to present to hospital with critical COVID-19. The association between COVID-19 severity and the ABO group may be due to the development of neutralizing antibodies against N-linked glycans that extensively cover SARS-CoV-2 spike protein or due to the stabilization of the von Willebrand factor including a one nucleotide deletion in exon 6 (codon 87) determining O allele. Four SNPs at residues 176, 235, 266, and 268, which switch enzyme function from A transferase to B transferase activity, may also contribute to the current findings (12, 40, 64).

Blood groups are also linked to several other diseases, including cardiovascular diseases and pulmonary thromboembolism, which have been identified as one of the main complications in COVID-19 disease progression (21, 65). The ABO blood type trait reflects polymorphisms within the *ABO* gene. The *ABO* gene is associated with a number of other traits including risk factors for COVID-19 morbidity and mortality. The genetic locus located within the *ABO* gene plays a role in hemoglobin concentration, hematocrit (66, 67), von Willebrand factor (68), myocardial infection (69), coronary artery disease (70), ischemic stroke (71), type 2 diabetes (72), and venous thromboembolism (73). In fact, one study recently reported an overall 24% cumulative incidence of pulmonary embolism in patients with COVID-19 pneumonia, of which 50% were the ICU patients (74, 75). Hence, this study does not limit itself to identify *ABO* links with COVID-19, but investigated comorbidities reported in a population sample that were admitted to hospitals in the UAE with confirmed SARS-CoV-2 infection. However, a similar finding has been demonstrated in this study, where specific risk alleles of SNPs were upregulated in the critical COVID-19 group.

Potential limitations of this study also need to be considered. Due to the pandemic associated lockdown over several months, we were unable to collect data on a large number of patients. Selection bias is a fundamental limitation of this study, so the association estimates are conditional on presentation to the hospital. Nonetheless, the methodology of the cross-sectional study minimized additional selection bias, with respect to the outcome of interest. Due to the geographical location of the Middle East region, there is substantial genetic admixture present within the population. However, to limit population stratification, 10 eigenvectors were adjusted for. An important limitation was the use of a GWAS arrays that were manufactured based on the Caucasian population and this limits the possibility of including targeted SNPs in the genome. To limit this error, imputation of genotypes for genetic variants that are untyped in the arrays increases the information provided by each microarray by accurately evaluating the evidence for association at genetic markers that are not directly genotyped. However, the imputed SNPs from previous studies did not reach significance suggesting that there may be specific population characteristics associated with COVID-19 severity. The inclusion of multiple data collection sites from across the country may have included cases that were not entirely homogeneous. It is possible that the criteria for hospitalization of patients with COVID-19 are different across the collection sites, thus measurement errors may exist in this study. Misclassification and ascertainment biases in the control group were limited by only selecting patients with COVID positive with the noncritical or critical cases.

## Conclusion

There is much to be done to understand the role of ABO and its association to COVID-19 severe phenotypes. The combination of genetic and serological evidence of the involvement of the ABO blood groups and *ABO gene* allelic associations with COVID-19 severity provides a unique opportunity to study host genomics to the interindividual phenotypic variability. By conducting a GWAS and extracting the SNPs in the *ABO* gene, we have provided further insight into the genetic mechanisms associated with COVID-19 disease severity and the possible link to allelic variants and COVID-19 critical phenotypes. Future study will address linking genomic data to electronic health records that can be leveraged to improve clinical management and lead to better patient outcomes.

## Data Availability Statement

The datasets presented in this study can be found in online repositories. The names of the repository/repositories and accession number(s) can be found in the article/Supplementary Material.

## Ethics Statement

This study was approved by the Abu Dhabi Health COVID-19 Research Ethics Committee (DOH/DQD/2020/538), Dubai Scientific Research Ethics Committee (DSREC-04/2020_09), and SEHA Research Ethics Committee (SEHA-IRB-005). The patients/participants provided their written informed consent to participate in this study.

## United Arab Emirates COVID-19 Collaborative Partnership

Juan Acuna, Khalifa University of Science and Technology, Abu Dhabi, United Arab Emirates; Eman Alefishat, Khalifa University of Science and Technology, Abu Dhabi, United Arab Emirates; Ernesto Damiani, Khalifa University of Science and Technology, Abu Dhabi, United Arab Emirates; Samuel F. Feng, Khalifa University of Science and Technology, Abu Dhabi, United Arab Emirates; Andreas Henschel, Khalifa University of Science and Technology, Abu Dhabi, United Arab Emirates; Abdulrahim Sajini, Khalifa University of Science and Technology, Abu Dhabi, United Arab Emirates; Ahmed Yousef, Khalifa University of Science and Technology, Abu Dhabi, United Arab Emirates; Bassam Ali, United Arab Emirates University, Al Ain, United Arab Emirates; Hiba Alhumaidan, Sheikh Khalifa Medical City and SEHA, Abu Dhabi; Hala Imambabaccus, Sheikh Khalifa Medical City and SEHA, Abu Dhabi, United Arab Emirates; Amirtharaj Francis, Sheikh Khalifa Medical City and SEHA, Abu Dhabi, United Arab Emirates; Stefan Weber, Sheikh Khalifa Medical City and SEHA, Abu Dhabi, United Arab Emirates; Mohammad Tahseen Al Bataineh, University of Sharjah, Sharjah, United Arab Emirates; Rabih Halwani, University of Sharjah, Sharjah, United Arab Emirates; Rifat Akram Hamoudi, University of Sharjah, Sharjah, United Arab Emirates; Abdulmajeed Al Khajeh, Dubai Health Authority, Dubai, United Arab Emirates; Laila Salameh, Dubai Health Authority, Dubai, United Arab Emirates.

## Author Contributions

HAls and GT conceived the project to study the role of the virus and host in COVID-19 in the United Arab Emirates. HJ and HAlH conceived the central research questions for the ABO data and initiated the first draft of the manuscript. MM, GD, and HJ analyzed and constructed the figures. BM, FA, MU, and NA were responsible for the recruitment of the patients and collecting data for the study. HJ, GT, EA, and HAls provided critical review during manuscript preparation. All authors on the primary list contributed to the data interpretation and critically reviewed the manuscript and approved the final manuscript for submission.

## Funding

This study was commissioned as part of a project to study the host cell receptors of coronaviruses funded by Khalifa University's CPRA grant (Reference Number 2020-004). The project was funded by internal funds provided by Khalifa University awarded to HAls.

## Conflict of Interest

The authors declare that the research was conducted in the absence of any commercial or financial relationships that could be construed as a potential conflict of interest.

## Publisher's Note

All claims expressed in this article are solely those of the authors and do not necessarily represent those of their affiliated organizations, or those of the publisher, the editors and the reviewers. Any product that may be evaluated in this article, or claim that may be made by its manufacturer, is not guaranteed or endorsed by the publisher.
