# Supplementary material for: Allelic Variants Within the ABO Blood Group Phenotype Confer Protection Against Critical COVID-19 Hospital Presentation
Source: Front Med (Lausanne). 2022 Jan 13;8:759648. doi: 10.3389/fmed.2021.759648 (PMC8793802; doi:10.3389/fmed.2021.759648)
Supplement: Supplementary file 1 [file Table_1.DOCX]

Supplementary Material

# Supplementary Table

Table 1. Stratification of blood group by age and BMI.

| Blood Group | Variables | | Non-Critical  N (%) | Critical  N (%) | *p*-value |
| --- | --- | --- | --- | --- | --- |
| A | Age | 1-29 | 14 (14.7%) | 1 (1.5%) | 0.001 |
|  |  | 30-38 | 23 (24.2%) | 9 (13.8%) |  |
|  |  | 39-49 | 30 (31.6%) | 17 (26.2%) |  |
|  |  | 50-85 | 28 (29.5%) | 38 (58.5%) |  |
|  | BMI | ≤18.5 | 3 (3.3%) | 0 (0.0%) | 0.073 |
|  |  | >18.5 to ≤ 24.9 | 29 (31.5%) | 14 (21.5%) |  |
|  |  | >24.9 to ≤ 29.9 | 36 (39.1%) | 23 (35.4%) |  |
|  |  | >29.9 | 24 (26.1%) | 28 (43.1%) |  |
| B | Age | 1-29 | 12 (15.6%) | 2 (3.5%) | <0.001 |
|  |  | 30-38 | 20 (26.0%) | 9 (15.8%) |  |
|  |  | 39-49 | 24 (31.2%) | 9 (15.8%) |  |
|  |  | 50-85 | 21 (27.3%) | 37 (64.9%) |  |
|  | BMI | ≤18.5 | 1 (1.3%) | 0 (0.0%) | 0.063 |
|  |  | >18.5 to ≤ 24.9 | 32 (41.6%) | 12 (21.1%) |  |
|  |  | >24.9 to ≤ 29.9 | 24 (31.2%) | 24 (42.1%) |  |
|  |  | >29.9 | 20 (26.0%) | 21 (36.8%) |  |
| AB | Age | 1-29 | 6 (26.1%) | 0 (0.0%) | 0.030 |
|  |  | 30-38 | 4 (17.4%) | 0 (0.0%) |  |
|  |  | 39-49 | 5 (21.7%) | 2 (16.7%) |  |
|  |  | 50-85 | 8 (34.8%) | 10 (83.3%) |  |
|  | BMI | ≤18.5 | 0 (0.0%) | 0 (0.0%) | 0.489 |
|  |  | >18.5 to ≤ 24.9 | 7 (31.8%) | 2 (16.7%) |  |
|  |  | >24.9 to ≤ 29.9 | 11 (50.0%) | 6 (50.0%) |  |
|  |  | >29.9 | 4 (18.2%) | 4 (33.3%) |  |
| O | Age | 1-29 | 27 (18.5%) | 1 (1.9%) | <0.001 |
|  |  | 30-38 | 53 (36.3%) | 3 (5.8%) |  |
|  |  | 39-49 | 30 (20.5%) | 11 (21.2%) |  |
|  |  | 50-85 | 36 (24.7%) | 37 (71.2%) |  |
|  | BMI | ≤18.5 | 3 (2.1%) | 1 (1.9%) | 0.676 |
|  |  | >18.5 to ≤ 24.9 | 37 (25.5%) | 10 (19.2%) |  |
|  |  | >24.9 to ≤ 29.9 | 64 (44.1%) | 22 (42.3%) |  |
|  |  | >29.9 | 41 (28.3%) | 19 (36.5%) |  |
